# Evaluation of dihydrotestosterone levels and total testosterone to dihydrotestosterone ratio with clinical symptoms and metabolic parameters in patients with polycystic ovary syndrome

**DOI:** 10.1186/s12902-025-02070-4

**Published:** 2025-11-13

**Authors:** Asal Ebrahimian, Afshin Moradi, Vahid Kheyri, Farzad Najafipour, Vahideh Sadra

**Affiliations:** 1 Student Research Committee, Tabriz University of Medical Sciences, Tabriz, Iran; 2 Present Address: Department of Endocrine Research Center, Imam Reza Hospital, Tabriz University of Medical Sciences, Golgasht Street, Tabriz, 51664 Iran

**Keywords:** Dihydrotestosterone (DHT), Total testosterone, Total testosterone to dihydrotestosterone ratio (TT/DHT), Polycystic ovary syndrome (PCOS), Metabolic parameters, Clinical symptoms, Androgens, Endocrine disorders

## Abstract

**Background:**

Polycystic ovary syndrome (PCOS) is a clinically heterogeneous endocrine disorder with lifelong health risks. Androgen excess, particularly testosterone, plays a key role in the pathophysiology of PCOS. Dihydrotestosterone (DHT) is an active metabolite derived from testosterone, and the total testosterone (TT) to DHT ratio is known as a potential marker of androgen metabolism, which can be used for evaluation of metabolic phenotype. This exploratory study aims to evaluate the association between DHT levels and TT/DHT ratio with the metabolic parameters and clinical features of PCOS patients.

**Methods:**

This cross-sectional study was conducted on 30 patients diagnosed with PCOS based on the Rotterdam 2003 criteria. Clinical, metabolic, and biochemical parameters were assessed. The association between DHT levels, TT/DHT ratio, and clinical as well as metabolic features was analyzed using ANOVA, Spearman correlation, and independent sample t-tests, with a significance threshold of *p* < 0.05.

**Results:**

The TT/DHT ratio was significantly lower in patients with regular menstruation compared to those with oligomenorrhea and amenorrhea (*p* < 0.05). A moderate positive correlation was found between DHT levels and BMI (*r* = 0.487, *p* < 0.05), while fasting blood sugar (FBS) was significantly correlated with the TT/DHT ratio (*r* = 0.376, *p* < 0.05). Patients with insulin resistance had a significantly higher TT/DHT ratio compared to those without insulin resistance (*p* < 0.05), whereas DHT levels did not differ significantly between these groups.

**Conclusion:**

The TT/DHT ratio could be a useful biomarker to identify PCOS patients with more severe metabolic issues.

**Supplementary Information:**

The online version contains supplementary material available at 10.1186/s12902-025-02070-4.

## Introduction

Polycystic ovary syndrome (PCOS) is a genetically complex and clinically heterogenous disorder of the endocrine system, which occurs in reproductive age women [1]. However, life-long health risks related to this syndrome can persist after menopause [2]. With increasing number of women with PCOS in the last decade, the global prevalence of PCOS is estimated about 9.2% (95% CI: 6.8–12.5%) [3]. The etiology of PCOS is unknown, and it is one of the most common causes of menstrual dysfunction, anovulatory infertility, and hyperandrogenism [4]. PCOS includes a broad spectrum of clinical features, including: oligomenorrhea, amenorrhea, hirsutism, alopecia, acne, acanthosis nigricans, and hyperandrogenism [5]. Functional ovarian hyperandrogenism is the most common cause of elevated androgen levels and related manifestations of this syndrome [6]. In addition, androgen excess plays a prominent role in the development of metabolic abnormalities such as obesity, resistance to insulin, hyperlipidemia, and liver dysfunctions in PCOS patients [7, 8]. These metabolic dysregulations increase the risk of PCOS patients developing type 2 diabetes mellitus, coronary heart disease (CHD), atherogenic dyslipidemia, depression, anxiety, and endometrial cancer [9–11]. The Rotterdam criteria is the most commonly used criteria, which establishes a diagnosis when at least two out of three of the following are present, provided that other conditions mimicking PCOS have been excluded: Oligo-anovulation, clinical and/or biochemical signs of hyperandrogenism, and polycystic ovaries (≥ 12 follicles measuring 2–9 mm in diameter and/or an ovarian volume > 10 mL in at least one ovary) [12].

Total Testosterone (TT) affects metabolic parameters and it is known that increasing testosterone is associated with less body fat, elevated insulin sensitivity, and decreased glycemia. Moreover, TT is negatively associated with the occurrence of Metabolic syndrome among adult women [13, 14]. Measurement of TT, as an androgen, is one of the diagnostic features of PCOS. However, by only measuring testosterone, hyperandrogenism might be missed in PCOS patients who have high levels of androstenedione but normal testosterone. Thus, it is important to measure other metabolites derived from steroid hormones [15].

Dihydrotestosterone (DHT) is an active metabolite derived from testosterone via 5-alpha reductase and regarding their difference, DHT is more potent and binds to androgen receptor with higher affinity compared to Testosterone [16]. DHT levels, especially the total testosterone-to-DHT (TT/DHT) ratio, are used to diagnose 5-alpha-reductase deficiency is a key androgen in androgenetic alopecia, but serum DHT levels do not reliably diagnose the condition, especially in women. The key factor is how sensitive hair follicles are to DHT, which varies genetically [17]. Previous animal studies found that DHT regulates food intake and leads to obesity in female mice [18, 19]. The TT/DHT ratio is a potential marker of androgen metabolism and enzymatic activity and can be used as a biomarker for evaluation of metabolic phenotype [20].

In this exploratory study we aimed to assess the association between DHT levels and TT/DHT ratio with metabolic parameters and clinical features of PCOS patients. The potential benefit of finding these associations could help identify whether DHT and TT/DHT ratio can help detecting PCOS patients who are at higher risk of developing more severe metabolic symptoms.

## Methods

### Study design

This study was conducted in a cross-sectional analytical manner, adhering to the STROBE guidelines for cross-sectional studies.

### Setting

Thirty patients who visited the endocrine clinic of Imam Reza Hospital, Tabriz, Iran, from March 2019 to March 2020 and were diagnosed with PCOS were included in this study.

### Participants

#### Inclusion criteria

Patients diagnosed with PCOS based on Rotterdam 2003 criteria.

#### Exclusion criteria

Patients with systemic diseases, a drug history of oral contraceptives and anti-androgens (Spironolactone and Finasteride) within 3 months prior inclusion in this study, patients undergoing hormone replacement therapy, and pregnant patients were excluded.

#### Variables

Clinical information of 30 patients with PCOS was collected from their medical records, clinical examinations, and blood samples including: symptoms (presence of hirsutism, acnes, and androgenic alopecia, menstrual history, and fertility) and metabolic parameters (body mass index [BMI], fasting blood sugar [FBS], insulin levels and insulin resistance, lipid profile (total HDL-cholesterol, triglyceride). Blood samples were taken in the morning (after an overnight fast). For women with regular menstrual cycles, sampling was performed on fifth day of the follicular phase. For women with amenorrhea, blood samples were taken regardless of the menstrual cycle.

### Measurements

Morphology of ovaries was assessed by sonography following Rotterdam 2003 criteria. Hirsutism was confirmed according to Ferriman-Gallwey score ≥ 8 [21]. Severity of Hirsutism, acnes and alopecia was classified into mild, moderate, and severe. Menstrual disorders were defined based on oligomenorrhea (menstrual cycles that are irregular and occur less frequently than normal, typically longer than 35 days apart or fewer than 4 to 9 cycles per year [22].) and amenorrhea (primary amenorrhea is defined as having no history of menstruation by the age of 15 years or 3 years after thelarche; secondary amenorrhea is defined as the absence of menses for ≥ 3 months in a woman with previously regular menstrual cycles or ≥ 6 months in any woman with at least one previous spontaneous menstruation [23]). Fertility status was recorded based on fertile (has been pregnant before), infertile (diagnosed infertility), and single/unknown (fertility status is unclear and no history of pregnancy). BMI was calculated by dividing the patient’s weight in kilograms by the square of their height in meters. FBS, total HDL-cholesterol, and triglyceride were all evaluated using Pars Azmun (Iran) kits. The assays for total cholesterol, HDL-cholesterol, and triglyceride were based on enzymatic colorimetric methods utilizing enzymes such as Cholesterol Oxidase (CHOD), with Phenol-Aminophenazone (PAP) serving as a chromogenic reagent for color development. Insulin resistance was verified in accordance with homeostatic model assessment for Insulin resistance (HOMA-IR) score ≥ 2.5. HOMA-IR is obtained by multiplying FBS in mg/dL and fasting serum Insulin levels in µIU/ml and dividing the result by 405.

Hormonal measurements were performed using ELISA and chemiluminescence immunoassay kits from the following companies: Prolactin (Pishtaz Teb, Iran), Estradiol, Dehydroepiandrosterone sulfate (DHEAS), and Insulin (Monobind, USA), Dihydrotestosterone (DHT) (Demeditec Diagnostics GmbH, Germany) and total serum Testosterone by chemiluminescence immunoassay on the Abbott Architect system.

### Statistical methods

For statistical analysis, SPSS version 25 was used. To assess the independent quantitative ratio variable (serum DHT level and the ratio of TT/DHT), nominal dependent quantitative variable (fertility status), ANOVA test was used. For normality distribution, the Shapiro-Wilk test was applied. For assessing quantitative ratio variable and dependent ordinal quantitative variable (menstruation, hirsutism, acne, androgenic alopecia) ANOVA test was used. The correlation between independent quantitative ratio (serum Dihydrotestosterone level and the ratio of TT/DHT) and dependent quantitative variables (FBS, serum Insulin, total serum cholesterol, serum triglyceride, HDL, and BMI) was evaluated by Spearman correlation coefficient and the correlation between serum Dihydrotestosterone level, the ratio of total Testosterone to serum DHT and Insulin resistance, independent sample t-test was used. In this study, a p-value of lesser than 0.05 was considered significant.

## Results

In this study, 30 patients with PCOS diagnosis were included. The mean age of patients was 30 ± 6.6. Among 30 patients, 11 (36.7%) of them had mild acne, 14 (46.7%) had moderate acne, and 5 (16.7%) of them had severe acne. Nine (30%) patients had mild alopecia, 15 (50%) had moderate alopecia, and six (20%) had severe alopecia. Hirsutism was presented mildly in six (20%) patients, moderately in 18 (60%) patients and severely in six (20%) patients (Table 1).

**Table 1:** Severity of Acne, Alopecia, hirsutism among patients with PCOS

severity	Acne	Alopecia	Hirsutism
Mild	11(36.7%)	9 (30%)	6 (20%)
Moderate	14 (46.7%)	15 (50%)	18 (60%)
Severe	5 (16.7%)	6 (20%)	6 (20%)

In terms of fertility status, nine (30%) patients were infertile, nine (30%) patients were fertile and fertility status of remaining 12 (40%) patients was unknown. Regarding menstruation, six (20%) patients had regular menstrual cycle, 18 (60%) had oligomenorrhea and six (20%) had amenorrhea (Table 2).

**Table 2:** Menstruation and fertility distribution

	Absolute Frequency (percentage)
Menstruation	
Regular	6 (20%)
Oligomenorrhea	18 (60%)
Amenorrhea	6 (20%)
Total	30 (100%)
Fertility	
Fertile	9 (30%)
Infertile	9 (30%)
Single/unknown	12 (40%)
Total	30 (100%)

Table 3 includes the descriptive statistics of metabolic parameters and other quantitative variables. According to Shapiro-Wilk test, the variables had normal distribution (*P*-value > 0.05).

**Table 3:** Metabolic phenotype and variable

Variable	Mean	Standard Deviation	*P*-value
Age (years)	28.90	5.63	0.72
BMI (kg/m²)	30.03	5.23	0.20
FBS (mg/dL)	93.13	9.42	0.17
Insulin (µIU/mL)	20.81	11.19	0.14
HOMA-IR	4.98	3.05	0.11
Triglyceride (mg/dL)	192.87	95.94	0.20
Total Cholesterol (mg/dL)	194.33	23.57	0.13
HDL (mg/dL)	37.29	7.51	0.20
DHEAS (µg/dL)	255.36	113.75	0.20
Prolactin (ng/mL)	23.40	11.60	0.20
DHT (pg/mL)	362.60	84.77	0.20
Total Testosterone (ng/mL)	0.50	0.24	0.20
(TT/DHT) * 1000	1.49	0.83	0.20

*BMI* Body Mass Index, *FBS* Fasting Blood Sugar, *HOMA-IR* Homeostatic Model Assessment of Insulin Resistance, *HDL* High-Density Lipoprotein, *DHEAS* Dehydroepiandrosterone sulfate, *DHT* Dihydrotestosterone, *TT/DHT* Total testosterone to Dihydrotestosterone ratio

The mean BMI of patients was 30.3 ± 5.63. Fifteen patients (50%) were classified as obese (BMI ≥ 30 kg/m²), 13 patients (43%) were overweight (BMI 25.0–29.9 kg/m²), and the remaining two patients (7%) had normal weight (BMI 18.5–24.9 kg/m²).

Twenty (66.7%) patients had resistance to insulin (HOMA-IR ≥ 2.5), while insulin resistance was absent in seven patients. Due to lack of data (insulin level) of the remaining three patients, their insulin resistance status was unknown (Table 4).

**Table 4:** Distribution of insulin resistance

Insulin Resistance	Absolute Frequency	Percentage (%)
Present	20	66.7
Absent	7	23.3
Missing data	3	10.0
Total	30	100

Comparison of three subgroups of fertility status using one-way ANOVA, didn’t show significant differences in DHT (F [2, 24] = 0.78, P-value > 0.05) and TT/DHT (F [2, 24] = 0.419, P-value > 0.05) levels (Supplementary Material Table S1).

For assessing the correlation between serum DHT levels and menstruation status, a one-way ANOVA was performed, and no significant differences were observed between patients with regular menstruation, oligomenorrhea and amenorrhea (F [2, 24] = 2.34, P-value >0.05). However, the difference in TT/DHT among three groups was significant (F [2, 24] = 4.43, P-value < 0.05) (Supplementary Material Table S1).

For multiple comparisons between different subgroups of menstruation status in terms of TT/DHT, LSD test was used. TT/DHT ratio was significantly lower (P-value < 0.05) in patients with regular menstruation (0.79 ± 0.48) compared to patients with oligomenorrhea (1.53 ± 0.72) and amenorrhea (2.07 ± 0.99). Nevertheless, it is worth mentioning that there wasn’t any significant difference in TT/DHT ratio between oligomenorrhea and amenorrhea groups (P-value > 0.05) (Supplementary Material Table S2).

The correlation between levels of DHT, TT/DHT ratio and severity of acne was assessed by means of ANOVA method. Levels of DHT and TT/DHT weren’t significantly different among patients with mild (TT/DHT: 1.42 ± 0.60, DHT: 378.90 ± 92.14), moderate (TT/DHT: 1.46 ± 1.00, DHT: 346.14 ± 92.53) and severe (TT/DHT: 1.49 ± 0.83, DHT: 372.80 ± 35.09) acne. (DHT: F [2, 24] = 0.49, P-value > 0.05, TT/DHT: F [2, 24] = 0.25, P-value > 0.05) (Supplementary Material Table S1).

To assess variation of alopecia severity among differing levels of DHT and TT/DHT, ANOVA method was used. Results showed that, among patients with mild (TT/DHT: 1.18 ± 0.52, DHT: 406.33 ± 107.34), moderate (TT/DHT: 1.65 ± 0.96, DHT: 338.13 ± 78.26) and severe (TT/DHT: 1.57 ± 0.85, DHT: 358.16 ± 28.81) alopecia, there wasn’t a significant correlation and difference in levels of DHT and TT/DHT. (DHT: F [2, 24] = 1.95, P-value > 0.05, TT/DHT: F [2, 24] = 0.93, P-value > 0.05) (Supplementary Material Table S1).

The correlation between level of DHT, TT/DHT ratio was assessed, and there was no significant association between DHT levels and TT/DHT ratio among groups with different hirsutism severity (Mild hirsutism: DHT = 368 ± 102.94, TT/DHT = 1.46 ± 0.78; Moderate hirsutism: DHT = 356.83 ± 75.08, TT/DHT = 0.80 ± 1.21; Severe hirsutism: DHT = 374.5 ± 107.66, TT/DHT = 1.00 ± 1.86) (Supplementary Material Table S1).

Results of Spearman correlation test between level of DHT, TT/DHT ratio and metabolic parameters showed that there was significant and moderate correlation between DHT level and BMI (*r* = 0.487, p-value < 0.05) and FBS was significantly correlated with TT/DHT ratio (*r* = 0.376, p-value < 0.05). There were no statistically significant correlations between DHT levels, TT/DHT ratio, Insulin, triglycerides, total cholesterol, HDL, prolactin, and DHEAS (p-value > 0.05) (Table 5).

**Table 5:** Spearman correlation between DHT, TT/DHT and variables

Variable	Correlation with DHT		Correlation with TT/DHT	
	Correlation Coefficient	p-value	Correlation Coefficient	p-value
FBS (mg/dL)	−0.267	0.157	0.376	0.041*
Insulin (µIU/mL)	0.037	0.85	0.163	0.42
Triglyceride (mg/dL)	0.015	0.944	0.233	0.274
Total Cholesterol (mg/dL)	0.276	0.192	0.108	0.615
HDL (mg/dL)	−0.006	0.978	−0.177	0.408
BMI (kg/m²)	0.487	0.006*	−0.344	0.06
DHEAS (µg/dL)	0.082	0.67	0.057	0.76
Prolactin (ng/mL)	−0.336	0.87	0.289	0.14
Fertility	−0.222	0.238	0.109	0.565
Acne	−0.035	0.854	0.085	0.655
Alopecia	−0.229	0.223	0.195	0.302
Hirsutism	0.018	0.924	0.219	0.244
HOMA-IR	−0.033	0.870	0.206	0.302
Age (years)	0.327	0.078	−0.248	0.187

*BMI* Body Mass Index, *FBS* Fasting Blood Sugar, *HOMA-IR* Homeostatic Model Assessment of Insulin Resistance, *HDL* High-Density Lipoprotein, *DHEAS* Dehydroepiandrosterone sulfate, *DHT* Dihydrotestosterone, *TT/DHT* Total testosterone to Dihydrotestosterone ratio

*P-value: significant

Results of independent samples t-test showed no significant difference in DHT levels between the group without insulin resistance and the group with insulin resistance (p-value > 0.05). However, the TT/DHT ratio was significantly lower in the group without insulin resistance compared to the group with insulin resistance (t(23.95) = −2.57, *P*-value < 0.05) (Supplementary Material Table S3).

## Discussion

PCOS is the most common endocrine disorder among women of reproductive age with many clinical manifestations [25]. TT is one the most commonly elevated clinical biomarkers in PCOS patients. A meta-analysis by Patil et al., indicated that free androgen index (FAI), a ratio of total testosterone to sex hormone binding globulin (SHBG), is higher in PCOS patients than in controls [26]. Furthermore, TT/DHT ratio is higher in women who have both PCOS and metabolic syndrome [20].

In this study, the prevalence of acne, alopecia, and hirsutism was high among PCOS patients. Despite this, we emphasize the fact that our analysis did not show a significant difference in DHT levels and TT/DHT ratio across different severities of these symptoms. Similarly, a study conducted by Quinn Met al., which included 254 PCOS patients, showed that hyperandrogenism (testosterone, total testosterone) among PCOS patients was not significantly more common in individuals with androgenetic alopecia (AGA) compared to those without AGA in PCOS patients [27]. These findings suggest that androgens may not be the sole factor responsible for the severity of clinical manifestations in PCOS. It is important to note that the lack of clear associations could be due to the relatively small sample size in our study. Larger studies may uncover subtle patterns, such as slightly higher DHT levels in patients with more severe symptoms, that are not evident here. Therefore, while our data do not show a strong link between androgen levels and PCOS symptom severity, we cannot definitively exclude such effect.

The prevalence of infertility in PCOS patients is reported to vary between 70 and 80%, with a 15-fold higher risk compared to women without PCOS [28]. In this study, the prevalence of infertility was 30%, and only 30% of patients were fertile. The status of 12 patients were unknown. The analysis showed that there is no significant correlation between DHT levels, TT/DHT ratio and infertility.

Regarding menstruation, patients with regular menstruations had significantly lower TT/DHT ratio compared to patients with oligomenorrhea and amenorrhea. However, TT/DHT ratio, was not significantly different between oligomenorrhea and amenorrhea groups. Consistent with our findings, a study by Ayala et al., reported that those with regular menstrual cycles have significantly lower levels of TT and DHT [29]. Additionally, a study by Atukorala et al., highlighted the importance of understanding fluctuations in testosterone levels during the menstrual cycle to establish more accurate reference ranges for clinical assessments [24].

The association between DHT levels and metabolic parameters was also investigated. We found a significant positive correlation between DHT and BMI in PCOS patients, indicating that individuals with higher BMI had higher DHT levels. However, DHT was not significantly correlated with triglyceride, total cholesterol, HDL, FBS, serum insulin, Prolactin and DHEAS levels. In the investigation of the association between TT/DHT and the metabolic parameters of PCOS patients, a significant correlation was found between FBS and TT/DHT, and this ratio was higher in those with higher FBS levels. Furthermore, the TT/DHT ratio was significantly higher in the group with insulin resistance. However, there wasn’t a significant correlation between DHT and insulin resistance.

In a study by Moran et al., they reported that PCOS patients with higher testosterone levels suffer from more adverse metabolic profile including greater total and abdominal obesity and insulin resistance [30]. Shah et al., found that TT/DHT ratio was significantly higher in PCOS patients but, the difference for DHT levels was not significant. They reported that, PCOS patients had significantly higher TT, free androgen index (FAI) and lower sex hormone binding globulin (SHBG) compared to controls. Moreover, the TT/DHT ratio was significantly higher in PCOS patients with impaired glucose tolerance (IGT) and metabolic syndrome, suggesting that TT/DHT may serve as a predictor of adverse metabolic outcomes in PCOS [31]. Ambroziak et al., also reported higher mean TT and androstenedione levels were in PCOS patients compared to controls. Moreover, Mean TT/DHT ratio was significantly higher in PCOS group compared to controls and it was correlated with BMI, waist circumference, %fat, as well as with insulin levels and resistance. However, the association between TT/DHT ratio and unfavorable metabolic parameters was also seen in controls and TT/DHT ratio, was found to be correlated with a worse metabolic profile not only in PCOS patients, but also in healthy women [32].

Münzker et al., showed significantly higher levels of TT, free testosterone, free DHT and TT/DHT ratio in PCOS patients compared to healthy controls, but they didn’t find a difference for total DHT levels. They reported a strong link between high TT/DHT ratio and adverse metabolic phenotype in PCOS patients and in PCOS patients alone, the TT/DHT ratio was significantly higher in obese patients and patients with metabolic syndrome, impaired glucose tolerance or insulin resistance. Our results align with these findings, supporting the role of the TT/DHT ratio in correlating with higher FBS and insulin resistance and its potential contribution to metabolic syndrome development [33]. Furthermore, Münzker et al., found that higher BMI was linked to lower DHT in PCOS. In contrast, our study results indicate a positive relationship between DHT and BMI, with patients who had higher BMI also having higher DHT levels. This discrepancy may stem from differences in sample size and DHT measurement methods; Münzker et al., used the gold standard method (LC-MS/MS), while our study used ELISA, and Münzker et al., included 275 patients while our study included 30 PCOS patients. Earlier animal studies found that mice treated with DHT had higher BMI due to enhanced food intake [18, 19]. The conflicting results between animal and human studies, as well as inconsistencies among different studies, highlight the importance of this issue. Most of the mentioned studies didn’t investigate the association between clinical symptoms such as alopecia, acnes, hirsutism fertility and menstruation status with DHT and TT/DHT ratio, whereas in the present study, this has been evaluated and among clinical symptoms, a strong correlation was only found between menstrual status and TT/DHT ratio and other symptoms weren’t significantly correlated with TT/DHT ratio and DHT levels. It is important to mention small sample size in our study, which cannot emphasize this correlation and further studies with large sample size are needed.

This study has several important limitations. First, the small sample size restricted statistical power, particularly in detecting moderate effects and conducting subgroup analyses. Consequently, some associations may have gone undetected, and several subgroup comparisons did not reach statistical significance. While this might reflect true lack of association, it is also possible that the sample size was insufficient to identify weaker effects. Additionally, the limited sample may not fully represent the clinical and phenotypic diversity of PCOS patients, thereby limiting the generalizability of our findings. Future studies with larger sample size and more diverse cohorts are necessary to validate and extend these results.

Second, multiple statistical tests were performed without correction for multiple comparisons due to the small sample size. Applying strict adjustments (e.g., Bonferroni correction) would further reduce power and increase the risk of false negatives. Thus, some marginally significant findings should be interpreted cautiously, as they may represent type I errors.

Third, we did not adjust for potential confounders such as age or BMI, which could influence hormone levels and metabolic parameters. The absence of multivariate or partial correlation analyses, limited by sample size, precludes determination of whether observed relationships such as between the TT/DHT ratio and fasting glucose are independent of other variables. Consequently, no causal or independent associations can be inferred from our data.

Fourth, the cross-sectional design restricts our ability to draw causal inferences between DHT levels, TT/DHT ratio, and PCOS clinical or metabolic features, since all variables were assessed simultaneously. Nonetheless, this design was appropriate for the initial exploratory aim of identifying potential associations that warrant further investigation through longitudinal studies.

Fifth, DHT was measured using an ELISA assay, which, while suitable for larger samples, may have limited sensitivity and specificity at the low hormone concentrations typical of female serum. Although the average DHT level in our cohort was above the assay’s detection limit, potential measurement error cannot be excluded. Future studies employing gold-standard techniques such as liquid chromatography-tandem mass spectrometry (LC-MS/MS) are recommended to improve the accuracy of steroid hormone quantification.

Finally, the absence of a healthy control group limits our ability to compare androgen and metabolic parameters against a baseline and may weaken the interpretability of our findings. Including controls in future research would provide a clearer understanding of how these measures differ between PCOS patients and unaffected populations.

## Conclusions

We found that Total Testosterone/dihydrotestosterone (TT/DHT) is significantly correlated with fasting blood sugar (FBS) and this ratio is significantly different among PCOS patients regarding menstruation status and insulin resistance. Moreover, body mass index (BMI) of PCOS patients is significantly correlated with serum DHT levels. Therefore, the TT/DHT ratio could serve as a potential biomarker to identify PCOS patients with more severe metabolic disturbances, nevertheless, its clinical utility requires confirmation in larger studies.

## Supplementary Information

Supplementary Material 1

## Data Availability

The data that support the findings of this study are available on request from the corresponding author. The data are not publicly available due to privacy or ethical restrictions.

## Abbreviations

PCOS: Polycystic Ovary Syndrome
TT: Total Testosterone
DHT: Dihydrotestosterone
TT/DHT: Total Testosterone to Dihydrotestosterone
BMI: Body Mass Index
FBS: Fasting Blood Sugar
PAP: Phenol-Aminophenazone
CHOD: Cholesterol Oxidase
DHEAS: Dehydroepiandrosterone Sulfate
SHBG: Sex Hormone Binding Globulin
FAI: Free Androgen Index
AGA: Androgenetic Alopecia
IGT: Impaired Glucose Tolerance
