# Supplementary material for: Evaluation of dihydrotestosterone levels and total testosterone to dihydrotestosterone ratio with clinical symptoms and metabolic parameters in patients with polycystic ovary syndrome
Source: BMC Endocr Disord. 2025 Nov 13;25:263. doi: 10.1186/s12902-025-02070-4 (PMC12617002; doi:10.1186/s12902-025-02070-4)
Supplement: Supplementary file 1 — Supplementary Material 1 [file 12902_2025_2070_MOESM1_ESM.docx]

|  | **TT/DHT** | | | | | | **DHT levels** | | | | | |
| --- | --- | --- | --- | --- | --- | --- | --- | --- | --- | --- | --- | --- |
|  | **Source** | **Sum of Squares** | **df** | **Mean Square** | **F** | **P-value** | **Source** | **Sum of Squares** | **df** | **Mean Square** | **F** | **P-value** |
| **Fertility** | Between Groups | 0.662 | 2 | 0.331 | 0.419 | 0.602 | Between Groups | 5724.919 | 2 | 2862.460 | 0.785 | 0.466 |
|  | Within Groups | 19.404 | 27 | 0.719 |  |  | Within Groups | 196955.361 | 27 | 7294.643 |  |  |
|  | **Total** | **20.006** | **29** |  |  |  | **Total** | **208405.200** | **29** |  |  |  |
| **mensuration status** | Between Groups | 2.471 | 2 | 1.236 | 4.429 | 0.022 | Between Groups | 15396.711 | 2 | 7698.356 | 2.341 | 0.116 |
|  | Within Groups | 15.064 | 27 | 0.558 |  |  | Within Groups | 177611.778 | 27 | 6578.214 |  |  |
|  | **Total** | **20.006** | **29** |  |  |  | **Total** | **208405.200** | **29** |  |  |  |
| **Acne** | Between Groups | 0.779 | 2 | 0.367 | 0.183 | 0.252 | Between Groups | 7237.77 | 2 | 3618.888 | 0.486 | 0.621 |
|  | Within Groups | 19.639 | 27 | 0.727 |  |  | Within Groups | 201167.423 | 27 | 7450.645 |  |  |
|  | Total | 20.006 | 29 |  |  |  | Total | 208405.200 | 29 |  |  |  |
| **Alopecia** | Between Groups | 1.286 | 2 | 0.643 | 0.408 | 0.927 | Between Groups | 26310.633 | 2 | 13155.137 | 1.951 | 0.162 |
|  | Within Groups | 18.720 | 27 | 0.693 |  |  | Within Groups | 182094.567 | 27 | 66744.243 |  |  |
|  | Total | 20.006 | 29 |  |  |  | Total | 208405.200 | 29 |  |  |  |
| **Hirsutism** | Between Groups | 1.293 | 2 | 0.647 | 0.933 | 0.406 | Between Groups | 1623.200 | 2 | 811.600 | 0.106 | 0.900 |
|  | Within Groups | 18.713 | 27 | 0.693 |  |  | Within Groups | 206782.000 | 27 | 7658.593 |  |  |
|  | Total | 20.006 | 29 |  |  |  | Total | 208405.200 | 29 |  |  |  |

Table S1. Comparison between DHT levels and DHT/TT ratio with clinical features using one-way ANOVA,

Table S2

The LSD test for determining differences between various menstrual groups in terms of DHT/T.

| **Multiple Comparisons (LSD)** | | | | | | |
| --- | --- | --- | --- | --- | --- | --- |
| **(I) Menstruation** | **(J) Menstruation** | **Mean Difference (I-J)** | **Std. Error** | **P-value** | 95% Confidence Interval | |
|  |  |  |  |  | Lower Bound | Upper Bound |
| **Regular** | Oligomenorrhea | -0.73630* | 0.35211 | 0.046 | -1.4588 | -0.0138 |
|  | Amenorrhea | -1.27427* | 0.43125 | 0.006 | -2.1591 | -0.3894 |
| **Oligomenorrhea** | Regular | 0.73630* | 0.35211 | 0.046 | 0.0138 | 1.4588 |
|  | Amenorrhea | -0.53797 | 0.35211 | 0.138 | -1.2605 | 0.1845 |
| **Amenorrhea** | Regular | 1.27427* | 0.43125 | 0.006 | 0.3894 | 2.1591 |
|  | Oligomenorrhea | 0.53797 | 0.35211 | 0.138 | - 0.1845 | 1.2605 |

Table S3

Correlation between insulin resistance and DHT, TT/DHT

|  | | **DHT** | | **TT/DHT** | |
| --- | --- | --- | --- | --- | --- |
|  |  | **Mean ± Standard Deviation** | **p-value** | **Mean ± Standard Deviation** | **p-value** |
| **Insulin resistance** | **+** | 349.35 ± 72.61 | 0.57 | 1.66 ± 0.37 | 0.017 |
|  | **-** | 369.14 ± 94.16 |  | 1.03 ± 0.9 |  |
